# Food Safety, Security, Sustainability and Nutrition as Priority Objectives of the Food Sector

**DOI:** 10.3390/ijerph18158073

**Published:** 2021-07-30

**Authors:** António Raposo, Fernando Ramos, Dele Raheem, Ariana Saraiva, Conrado Carrascosa

**Affiliations:** 1 CBIOS (Research Center for Biosciences and Health Technologies), Universidade Lusófona de Humanidades e Tecnologias, Campo Grande 376, 1749-024 Lisboa, Portugal; 2 Pharmacy Faculty, University of Coimbra, Azinhaga de Santa Comba, 3000-548 Coimbra, Portugal; framos@ff.uc.pt; 3 REQUIMTE/LAQV, Rua Dom Manuel II, Apartado 55142, 4051-401 Oporto, Portugal; 4 Northern Institute for Environmental and Minority Law (NIEM), Arctic Centre, University of Lapland, 96101 Rovaniemi, Finland; braheem@ulapland.fi; 5 Department of Animal Pathology and Production, Bromatology and Food Technology, Faculty of Veterinary, Universidad de Las Palmas de Gran Canaria, Trasmontaña s/n, 35413 Arucas, Spain; ariana_23@outlook.pt (A.S.); conrado.carrascosa@ulpgc.es (C.C.)

**Keywords:** environmental health, food safety, food security, nutrition, sustainability

Food systems are at the center of global environmental, social, and economic challenges such as resource scarcity, ecosystem degradation, and climate change [1]. The current food systems are generating negative outcomes, such as land, water, and ecosystem degradation, biodiversity loss, excessive greenhouse gas emissions, persistent malnutrition and hunger, and are failing to eradicate poverty, particularly of rural populations in the global South [2]. The future food systems will have to provide food and nutrition security while facing unprecedented sustainability challenges: this underlines the need for a transition to more sustainable food systems. Taking into account these premises and considering the complexity of food systems, this Special Issue presents 23 papers published by researchers from 24 different countries all over the world, including Australia, Brazil, Canada, Cape Verde, China, Croatia, Finland, France, Greece, Iran, Italy, Israel, Korea, Malaysia, Malta, Morocco, Portugal, Russia, Spain, Sweden, Taiwan, the UK, the USA, and Vietnam.

Regarding the review articles included in this Special Issue, it is possible to find four works that address the following themes: the bulk sweetener maltitol, where the analytical determination methods, applications in the food industry, metabolism, and health impacts are discussed in depth [3]; the relevance of food naturalness for consumers, food security aspects, sustainability, and health impacts, focused on natural sweeteners [4]; biofilm concerns from many angles, including biofilm-forming pathogens in the food industry, biofilm disinfectant resistance, and biofilm identification methods [5]; how texture and rheology might be evaluated in the food industry with specific attention on dysphagia [6].

In terms of research articles, we can mention 17 relevant works that focus on different areas common to the objectives of this Special Issue, namely: the study of green food intake and social trust as mediators in the relationship between perceived consumer effectiveness and psychological wellbeing [7]; the investigations by Bogdanova et al. [8] focused on integrating many components of the reindeer food value chain in a multidisciplinary manner to promote indigenous peoples’ food sovereignty in Western Siberia’s Arctic zone, as well as reflections on the key issues of the COVID-19 pandemic; an empirical study from China analyzing the effects of epistemic and social trust on public acceptance of genetically modified food [9]. This research adds to our understanding of how trust influences the acceptance of emerging technologies, and it is crucial for risk-management practices. The quasi-experimental pilot study assessed the effects of a pilot community-based behavioral intervention on the home food environment in U.S. [10]. In Italy, the results of a culturally tailored dietary intervention in diabetic patients from North Africa and Bangladesh were investigated by Piombo et al. [11]. Chen and Lin [12] have investigated and constructed the important and appropriate factors for designing a green café ambiance and empirically analyzed indicators with high operability that are suitable for green café ambience design. Another study from China used exploratory spatial data analysis, a gravity center model, a spatial panel data model, and a geographically weighted regression model to examine the spatial–temporal characteristics in grain production and their influencing factors using climate and socioeconomic data from 1995 to 2018 [13]. The nutritional profile, phenolic composition, and biological properties of *Crepis vesicaria* L. subsp. *taraxacifolia* leaves were analyzed in a research conducted by Pedreiro et al. [14]. The study by Fideles et al. [15] looked at food insecurity among Brazilian Community restaurant food handlers and the factors that contribute to it. In the U.S., Ferrante et al. [16] designed a survey to look at how COVID-19 affects parents’ lifestyles (e.g., work, child care, grocery shopping), as well as current family food acquisition and food habits (e.g., cooking, restaurant use); Nemati et al. [17] conducted a study to evaluate the effects of using different levels of ginger powder on the productive performance, eggs quality, and blood parameters in laying Japanese quails; Professor Heesup Han and colleagues have investigated the impact of halal food performance, which includes criteria such as availability, health/nutrition, accreditation, and cleanness/safety/hygiene, on Muslim traveler retention in a non-Islamic destination [18]. Evans et al. [19] investigated how surface characteristics (chemistry and topography) and cleaning direction affected the removal of bacteria and meat exuded from surfaces. The amounts of Al, Cd, Cr, Ni, Pb, and Sr in frequently consumed cereals and cereal-based products were determined in a study conducted in the Cape Verde Islands, and the risk associated with them was analyzed [20]. The study about the Indigenous community perspectives of food security, sustainable food systems, and strategies to enhance access to local and traditional healthy food for partnering Williams Treaties First Nations (Ontario, Canada) can be used to develop Indigenous community-based projects and initiatives aimed at improving food security, establishing more sustainable food systems, and achieving food sovereignty [21]; Wang et al. [22] investigated the effects of ecological compensation, capital endowment, and ecological cognition on the adoption of environmentally friendly technology by farmers.

On 25 November 2020, Professor Lluís Serra-Majem and his co-workers published an important Update of the Pyramid of the Mediterranean Diet, considering sustainability and focusing on environmental concerns [23]. This work has more strongly emphasized the lower consumption of red meat and bovine dairy products, and the higher consumption of vegetables and locally grown eco-friendly plant foods as much as possible. This paper is already an Editor’s Choice article and promoted the publication of two pertinent commentary papers: a work carried out by Professor Maria Luz Fernandez and collaborators on highlights of current dietary guidelines around the world [24], and also a text produced by the well-known Professor Walter Willett, mentioning that “all countries can benefit by considering this updated Mediterranean Dietary Pyramid when developing their dietary guidelines and food systems” [25].

There is a need for a ‘food systems thinking’ that takes cognizance of the link between the safety of food, food security, nutrition, and health at both individual and planetary levels. Our food system needs to be sustainable, as alluded to by some of the authors in this Special Issue. The food sector will need to make necessary changes and respond to sustainability challenges in our current food system; these include the negative outcomes mentioned earlier [1,2], population growth, climate change, and pandemics. This transformation of the food system, whose goal is to help stakeholders better understand and manage the complex choices that affect the future of food systems and to accelerate progress toward the Sustainable Development Goals, has already been discussed in several fora on a global level and will culminate at the UN SDG Food System summit in New York, the USA, by September 2021 [26]. The topics addressed in this Special Issue are thought-provoking and worth considering by researchers, academics, policymakers, food processors, indigenous peoples, and other stakeholders.

Last but not the least, the editors believe that this Special Issue gives an important contribution in the form of essential ingredients of ‘food for thought’, when safety, sustainability, and nutrition are considered as priority objectives in the food sector.

## Data Availability

Not applicable.
